# Quorum sensing triggers the stochastic escape of individual cells from *Pseudomonas putida* biofilms

**DOI:** 10.1038/ncomms6945

**Published:** 2015-01-16

**Authors:** Gerardo Cárcamo-Oyarce, Putthapoom Lumjiaktase, Rolf Kümmerli, Leo Eberl

**Affiliations:** 1 Department of Microbiology, Institute of Plant Biology, University of Zürich, Zollikerstrasse 107, Zürich CH-8008, Switzerland; 2 Department of Microbial Evolutionary Ecology, Institute of Plant Biology, University of Zürich, Winterthurerstrasse 190, Zürich CH-8057, Switzerland

## Abstract

The term ‘quorum sensing’ (QS) is generally used to describe the phenomenon that bacteria release and perceive signal molecules to coordinate cooperative behaviour in response to their population size. QS-based communication has therefore been considered a social trait. Here we show that QS signals (*N*-acyl-homoserine lactones, AHLs) are stochastically produced in young biofilms of *Pseudomonas putida* and act mainly as self-regulatory signals rather than inducing neighbouring cells. We demonstrate that QS induces the expression of putisolvin biosurfactants that are not public goods, thereby triggering asocial motility of induced cells out of microcolonies. Phenotypic heterogeneity is most prominent in the early stages of biofilm development, whereas at later stages behaviour patterns across cells become more synchronized. Our findings broaden our perspective on QS by showing that AHLs can control the expression of asocial (self-directed) traits, and that heterogeneity in QS can serve as a mechanism to drive phenotypic heterogeneity in self-directed behaviour.

## Abstract (web summary)

Bacteria secrete signalling molecules (AHLs) to coordinate actions such as biofilm formation and the release of public goods, in a process called quorum sensing. Here, the authors show that AHLs are stochastically produced and control asocial (self-directed) traits in young biofilms of *P. putida*.

## Graphical abstract

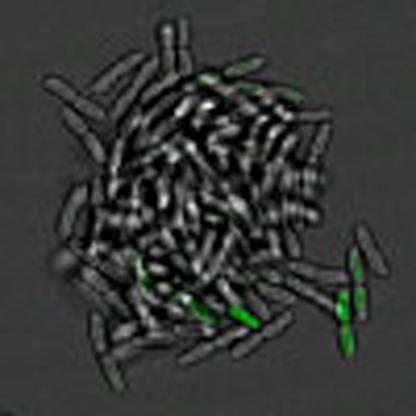

The term quorum sensing (QS) is used to describe the phenomenon that bacteria are capable of perceiving and responding to self-generated signal molecules to coordinate their behaviour at the group level^1^. The general consensus is that bacteria trigger the QS response only when their cell density has reached a certain threshold (the ‘quorum’), on which the expression of target genes is either activated or repressed. Among the various QS signal molecules identified to date, *N*-acyl-homoserine lactones (AHLs) have been investigated to the greatest extent^2^,^3^ and have been shown to control the expression of a large variety of traits, including bioluminescence, virulence, symbiosis, different forms of motility, biofilm formation, production of antibiotics and toxins, and conjugation^4^,^5^. Many AHL-controlled traits represent cooperative behaviours that can generate benefits to other cells in the local community^6^. Consequently, it has been suggested that QS has evolved to restrict the expression of costly cooperative behaviours to conditions, in which they are most beneficial, which is the case at high cell density^7^,^8^,^9^,^10^,^11^,^12^. However, recent studies in *Pseudomonas aeruginosa* have demonstrated that QS is more complex, because it also controls expression of a few cellular enzymes (that is, private goods)^10^,^13^,^14^,^15^. It has been suggested that co-regulation of public and private goods stabilizes cooperation, because it negates the selective advantage of cheating mutants, which exploit public goods without contributing to them^13^.

Here we studied the role of QS in biofilm formation in *P. putida* IsoF, a strain that has been isolated from the rhizosphere of a tomato plant^16^,^17^. In this strain, we have previously identified an AHL-dependent QS system, which is located on a genomic island, encoding PpuI, which directs the biosynthesis of the two AHLs 3-oxo-C10 and 3-oxo-C12 as major products; PpuR, the AHL receptor; and RsaL a repressor of *ppul*^17^. For a closely related strain (PCL1445), it is has been shown that the *ppu* system controls expression of a large non-ribosomal peptide synthethase (encoded by *psoA*, *psoB* and *psoC*), which directs the biosynthesis of the two cyclic lipopeptide biosurfactants putisolvin I and II^18^. The putisolvins were found to not only inhibit biofilm formation of *P. putida* PCL1445 but also to break down existing *P. aeruginosa* biofilms^19^. Previous work has shown that *P. putida* IsoF forms a flat and homogenous biofilm, whereas a *ppuI* mutant forms a structured biofilm with characteristic microcolonies and water-filled channel^17^. Although putisolvin production has not been demonstrated for *P. putida* IsoF, it has been hypothesized that QS-dependent expression of these biosurfactants could also affect biofilm structural development of this strain^18^,^20^.

In this study, we visualize AHL production at the single cell level to clarify the link between AHL-mediated QS, putisolvin production and biofilm development in *P. putida* IsoF. We show that at early stages of biofilm development, QS induces putisolvin production, which gives rise to a fraction of motile cells that leave the microcolony on their own. This asocial motility is possible because: (a) AHL signal production is stochastically expressed in only a fraction of the cells in young biofilms; (b) AHL production in one cell does not induce AHL production in its neighbouring cells; and (c) putisolvins cannot be used by other cells, and therefore do not represent public goods that can be shared among cells. At a later stage of biofilm development, the AHL expression pattern is more compatible with the expected cross-induction of cells within microcolonies, which results in a mass movement of cells and a concomitant collapse of microcolonies, giving rise to an unstructured biofilm.

## Results

### QS triggers asocial motility in young biofilms of *P. putida*

**Figure 1:**
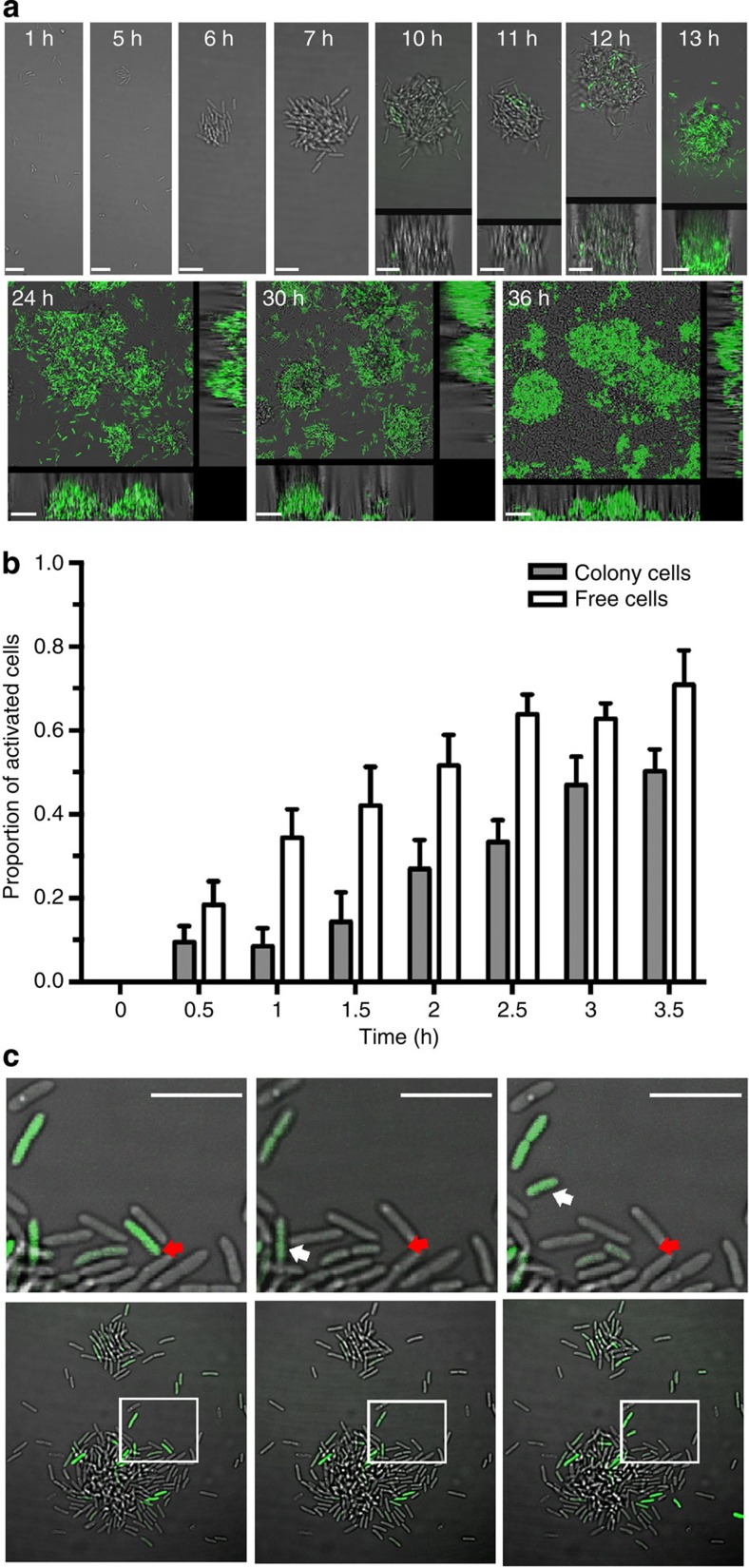
AHL production during biofilm development of *P. putida* IsoF. (**a**) Flow cells were inoculated with a low-density culture of *P. putida* IsoF carrying the GFP-based AHL sensor plasmid pRP4las. Green fluorescence, which is indicative of AHL production, and biofilm formation was followed by confocal laser scanning microscopy. Although AHL expression was stochastic at early stages of biofilm formation, the expression pattern became more homogenous across cells in older biofilms. The large frames show the top view, whereas the right and lower frames show vertical sections through the biofilms. Scale bars, 1–13 h, 5 μm; 24–36 h, 20 μm. (**b**) Focusing on early stages of biofilm formation (between 8 and 14 h post inoculation), quantitative analysis from eight independent frames revealed that the proportion of AHL-activated cells was significantly higher among free cells than among cells within colonies. This pattern remained consistent over time. Time 0 was defined as 30 min before the first appearance of induced cells (mean: 10.5±0.8 h post inoculation). (**c**) Many of the AHL-induced cells became highly motile and moved out of the microcolony, as exemplified in pictures from left to right, which were taken at 30-min intervals. The upper panel shows a close-up of the region indicated in the lower panel. The red arrow points at a cell that left the microcolony and the white arrow indicates a cell that moved to the periphery of the microcolony. Scale bar, 5 μm.

To investigate the role of AHL-mediated QS in biofilm formation of *P. putida* IsoF at the single cell level, we transferred the green fluorescent protein (GFP)-based AHL sensor plasmid pRP4las (with stringently controlled copy number: two to three per cell) into the wild-type strain. To ensure that none of the cells is AHL-induced before inoculation into the flow-through chamber^21^, we grew the strains for at least five generations at a low cell density (OD_600_<0.3) in minimal medium supplemented with citrate as carbon source. Following inoculation into the flow-through chambers, we monitored the spatial and temporal production of AHLs during biofilm development under defined conditions^22^. In our setup, small microcolonies were formed within 6–8 h; yet, at this point no fluorescent cells were detectable. When microcolonies reached a population size of 23.3±18.6 bacteria, after 11.9±1.3 h, a small fraction of cells turned green fluorescent, indicating that they had triggered the production of AHL signal molecules (Fig. 1a). Surprisingly, however, these induced cells did not seem to stimulate AHL production in neighbouring cells within the colony as one would expect according to the generally accepted paradigm that QS is a regulatory mechanism that co-ordinates behaviour at the group level. To rule out artefacts potentially associated with the use of a plasmid-based AHL reporter, we integrated the AHL reporter cassette into the chromosome of *P. putida* IsoF. Using this single-copy AHL biosensor, we quantified the number of induced cells within and outside of microcolonies. This analysis revealed that free cells outside colonies were significantly more often induced than cells within colonies (Fig. 1b, linear mixed model (LMM): *t*_111_=5.68, *P*<0.0001). Although the frequency of induced cells significantly increased over time (LMM: *t*_111_=11.73, *P*<0.0001), it increased similarly among free and colony cells (LMM, no significant interaction between time and cell status (free versus colony): *t*_111_=1.14, *P*=0.26), showing that QS induction level was consistently higher among free cells. This pattern is compatible with a two-step non-coordinated process starting with stochastic expression of AHL, followed by induced cells becoming motile and independently leaving the microcolonies. Indeed, we observed that induced cells left the colonies and were either removed by the nutrient flow or re-attached to the glass surface in the void spaces between the microcolonies (Fig. 1c).

To further elucidate the heterogeneity in QS induction, we exposed early-stage biofilms to a saturating concentration of 3-oxo-C10-HSL (0.5 μM). We observed that the timing of QS induction was slightly advanced (1.5 h), but that the heterogeneity in induction remained (Supplementary Fig. 1). This supports the idea that there are two distinct subpopulations of QS-responsive and non-responsive cells, whereby cross-induction between the two subpopulations is limited, at least in early-stage biofilms. Our observation is reminiscent of previous findings by Pradhan and Chatterjee^23^, who demonstrated the presence of stable subpopulations of QS-responsive and non-responsive cells in *Pseudomonas syringae* and *Xanthomonas campestris*.

At later stages of biofilm growth (Fig. 1a), we noticed an increase of fluorescent cell clusters within the microcolonies, which might be the result of AHL-mediated cross-stimulation. These clusters continuously increased in size until the large majority of cells of the microcolonies showed green fluorescence (Fig. 1a; usually in >30-h-old biofilms). At this point the microcolonies suddenly collapsed as a consequence of a mass movement of cells. The resulting biofilm was unstructured and uniformly covered the glass surface as has been reported previously for mature IsoF biofilms^17^.

### Putisolvin is required for motility and biofilm collapse

**Figure 2:**
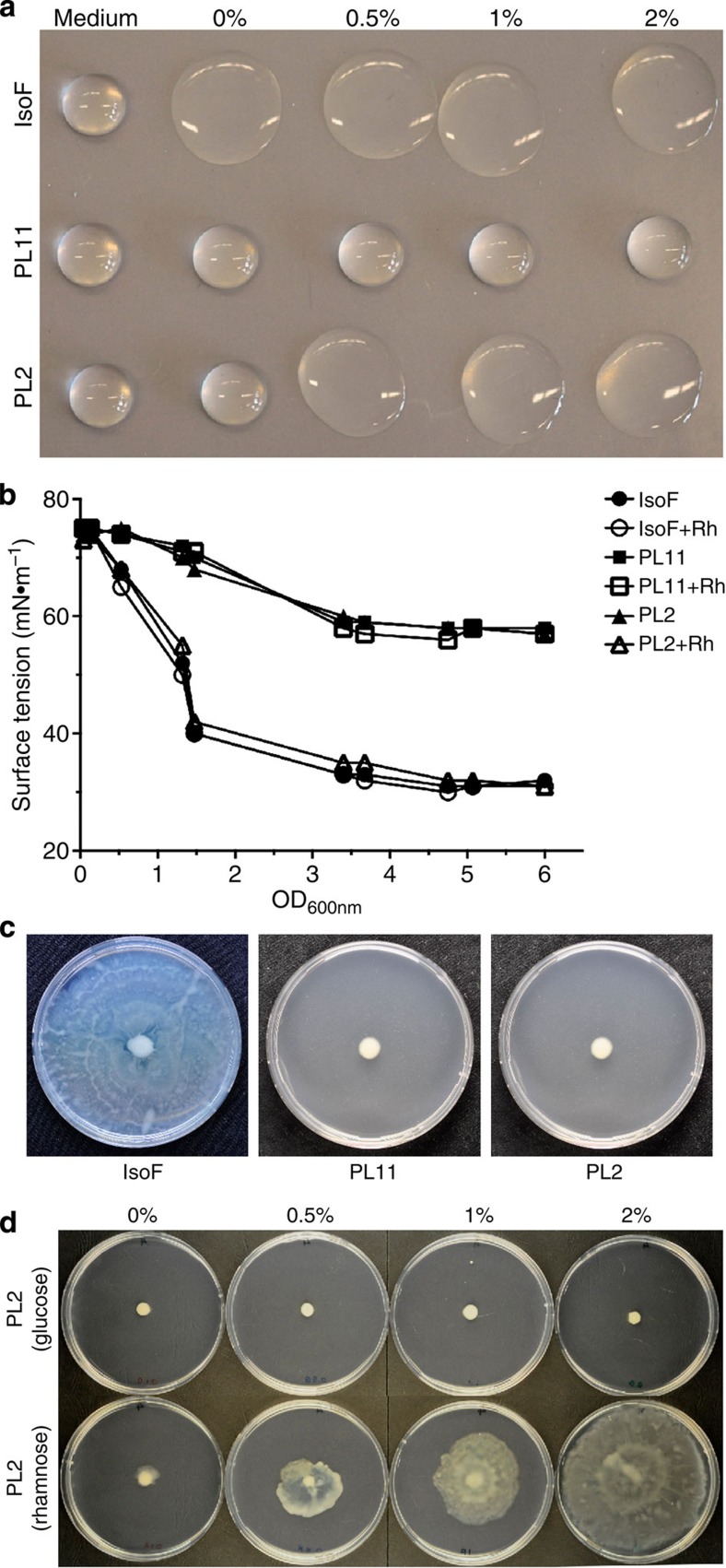
The production of biosurfactants and swarming motility in *P. putida* IsoF are dependent on the *pso* gene cluster. The wild-type IsoF, the *psoA* mutant PL11 and the conditional *psoA* mutant PL2 were grown in the absence or presence of rhamnose. (**a**) In drop-collapse assays, surface tension of supernatants of overnight cultures were found to be increased in PL11 compared with IsoF, but could be restored to wild-type level in PL2 on rhamnose supplementation. (**b**) Surface tensions of PL2, PL11 and IsoF in medium containing 1% or no rhamnose were quantified along the growth curve using the Du Nouy ring method. Results are representative of three independent experiments. (**c**) IsoF displays swarming motility in ABC medium, whereas PL11 and PL2 are impaired in swarming, because they do not produce putisolvin. (**d**) Swarming of the conditional *psoA* mutant PL2 was abolished on glucose plates, but was increasingly restored on plates supplemented with 0.5%, 1% or 2% rhamnose. Pictures were taken after 3 days of incubation.

In a next step, we aimed to better understand the mechanistic link between QS-heterogeneity and asocial motility in our system. As previous work had revealed a strong relationship between the production of putisolvin biosurfactants and biofilm formation in *P. putida* PCL1445 (refs 18, 19), we examined whether strain IsoF also harbours the putisolvin biosynthetic gene cluster, and whether asocial motility and biofilm collapse is linked to putisolvin production. By using a PCR approach combined with sequencing, we found that the entire *pso* gene cluster is present in strain IsoF and shows >99% DNA sequence identity with the *pso* locus of strain PCL1445. To investigate whether putisolvins act as a biosurfactant in the IsoF strain, we constructed the defined *psoA* knockout mutant PL11, as well as the conditional mutant PL2, in which the native promoter region of *psoA* has been replaced with the rhamnose-inducible *P*_*rhaB*_ promoter (Supplementary Fig. 2). In the absence of rhamnose, both mutant strains showed no surfactant activity in a simple drop-collapse assay (Fig. 2a). However, drop-collapsing activity of strain PL2 but not of PL11 could be restored when the medium was amended with at least 0.5% rhamnose. The Du Nouy ring method was used to measure surface tension of spent culture supernatants along the growth curve. Surface tension was found to reach a minimum when the cultures had an OD_600_ of ~2.0. Importantly, surface tension was found to be significantly reduced on rhamnose-induced putisolvin production (Fig. 2b). Next, we tested whether putisolvin is essential for swarming motility, as has been found to be the case for other biosurfactants in other bacterial species^24^. Indeed, when tested on swarming plates containing citrate as carbon source, the wild-type IsoF colonized the entire plate within 3 days, whereas no surface migration was observed for mutants PL2 and PL11 (Fig. 2c). However, in the case of PL2 swarming could be restored by amending the medium with 0.5% rhamnose (Fig. 2d), whereby the migration speed of the swarm colony was found to be proportional to the rhamnose concentration. These results demonstrate that putisolvin acts as a biosurfactant in *P. putida* IsoF and is essential for swarming.

**Figure 3:**
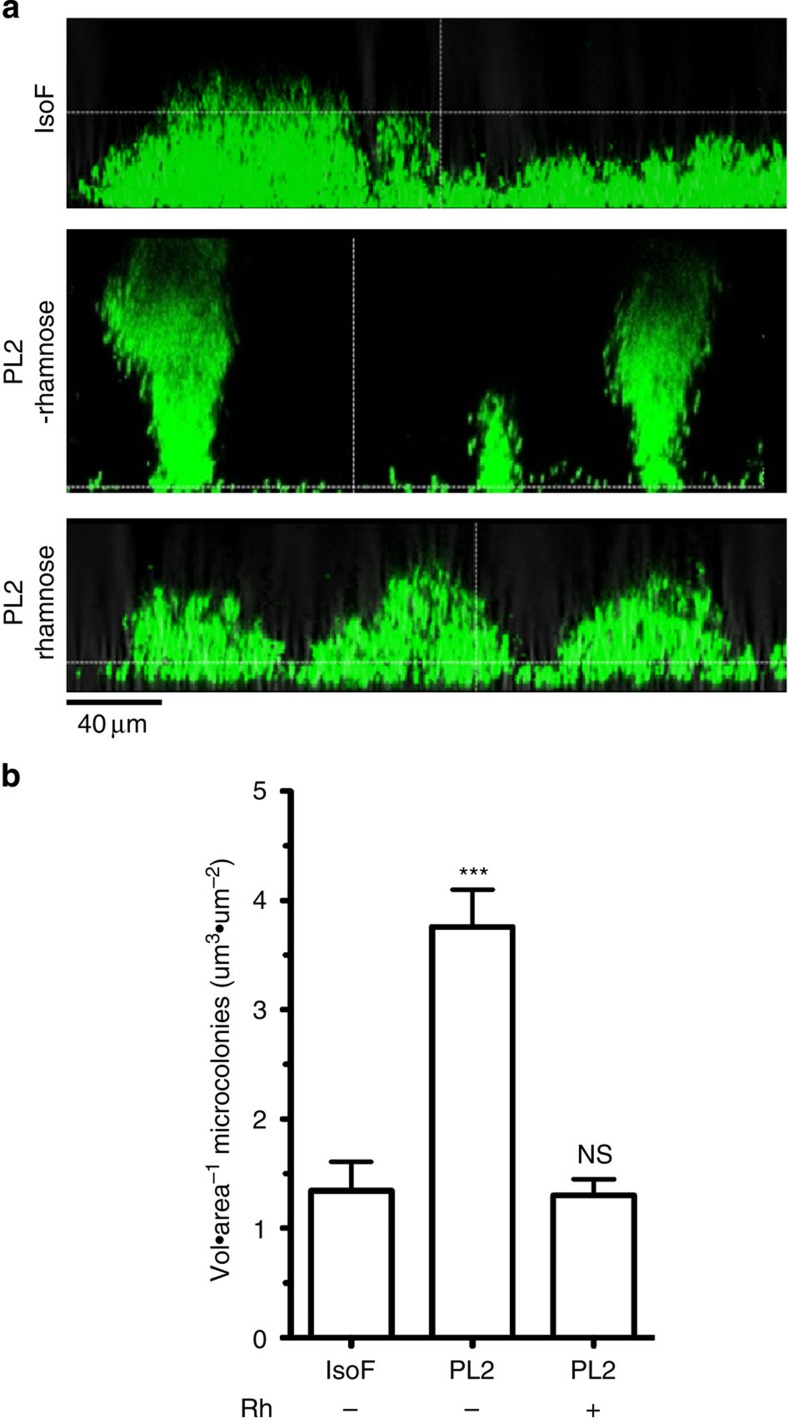
The role of putisolvin biosurfactants in biofilm structural development. Flow chambers were inoculated with *gfp*-tagged derivatives of the wild-type IsoF and the conditional *psoA* mutant PL2 in the absence or the presence of 0.2% rhamnose. (**a**) Vertical sections through the biofilms after 3 days show a flat biofilm structure for IsoF and PL2 amended with rhamnose, whereas PL2 without rhamnose forms biofilms that are dominated by towering microcolonies. (**b**) Three-dimensional parameter analysis of biofilm structures show that microcolonies of IsoF have significantly lower volume/area ratio than microcolonies of PL2. The wild-type phenotype could be restored in PL2 when adding rhamnose (Rh) to the medium. Mean values of three independent experiments are shown with s.e.m. (one-way analysis of variance; ***P<0.001; NS, not significant).

To test whether putisolvin is involved in biofilm collapse, we compared biofilm formation of the wild-type IsoF with the conditional *psoA* mutant PL2 in flow-through cells using AB minimal medium supplemented with 1 mM citrate. After 3 days of growth, the wild-type had formed a flat and unstructured biofilm with a low volume/area ratio, while the biofilm of mutant PL2 was dominated by large microcolonies, characterized by a threefold higher volume/area ratio and with only few cells colonizing the void space (Fig. 3). Addition of 0.2% rhamnose to the medium recovered the flat wild-type biofilm structure. These experiments demonstrate that putisolvins promote the colonization of the substratum by facilitating the movement of cells out of microcolonies.

### Putisolvin production is QS regulated

**Figure 4:**
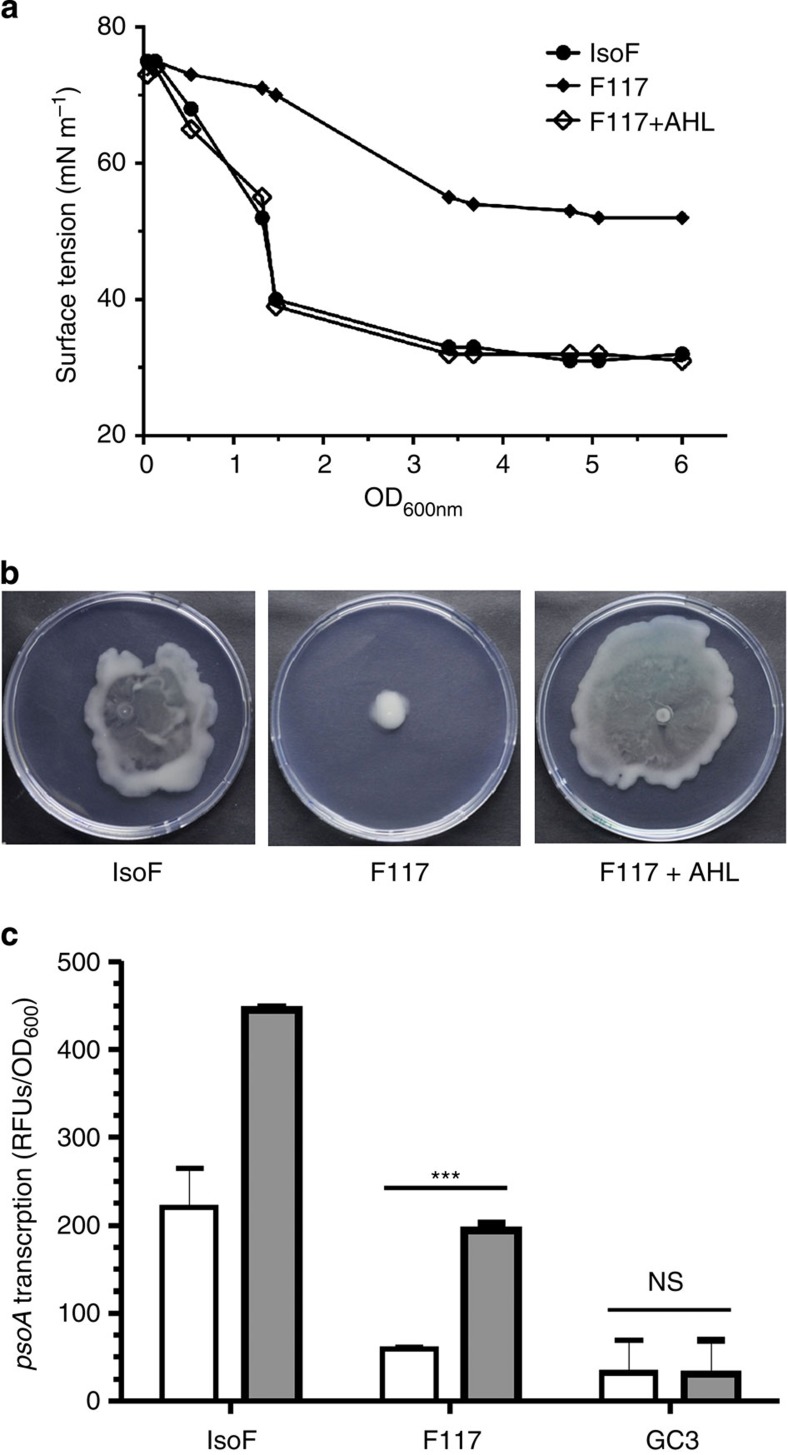
Putisolvin production and swarming motility are controlled by the *ppu* QS system in *P. putita* IsoF. The surface tension (**a**) and swarming motility (**b**) of the *ppuI* mutant F117 increased and decreased, respectively, but were restored to wild-type IsoF levels on supplementation with 5 μM of 3-oxo-C10-AHL. Surface tension of supernatants was measured by the Du Nouy ring method. Results are representative of three independent experiments. Swarming plates were photographed after 3 days of incubation. (**c**) When measuring the *psoA* promoter activity in ABC medium in the absence (open bars) or the presence of 5 μM of 3-oxo-C10-AHL (grey bars), *psoA* promoter activity could be restored to wild-type IsoF level in the *ppuI* mutant F117 (which does not produce AHL signals), but not in the *ppuR* mutant GC3 (which cannot respond to AHL signals). Mean values of three independent experiments are shown with s.d. (one-way analysis of variance; ***P<0.001; NS, not significant).

We further investigated whether AHL-mediated QS controls putisolvin production, thereby leading to the asocial motility phenotype and biofilm collapse observed in our single-cell experiments. Indeed, Dubern *et al*.^18^ have demonstrated that production of putisolvins in strain PCL1445 is regulated by the *ppuI-rsaL-ppuR* QS system. In agreement with this study, we found that a *ppuI* mutant of strain IsoF, named F117, was unable to swarm and spent culture supernatants of F117 cultures lacked surfactant activity (Fig. 4a,b). Both defects could be rescued by the addition of 5 μM 3-oxo-C10-AHL. These results strongly suggest that QS deficiency in F117 results in the abolishment of putisolvin production. To obtain more direct evidence for the link between QS and putisolvin production, we constructed a transcriptional fusion of the *psoA* promoter (triggering putisolvin synthesis) with *gfp* and transferred the resulting plasmid, pLUM1, into the wild-type IsoF, the *ppuI* mutant F117 (defective for AHL signal production) and the *ppuR* mutant GC3 (defective for responding to AHL signals). Measurements of GFP fluorescence revealed that *psoA* expression was strongly decreased in F117 and GC3, but could be restored to wild-type level for F117, but not for GC3, when the medium was supplemented with 5 μM 3-oxo-C10-HSL (Fig. 4c).

### The role of flagella for biofilm development of *P. putida*

**Figure 5:**
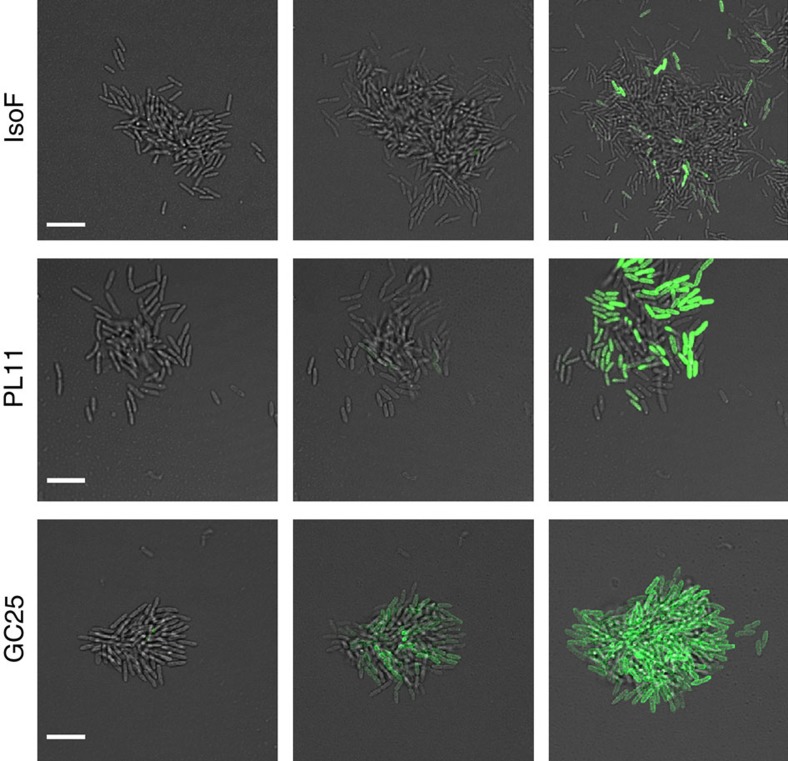
Activity of the *psoA* promoter during biofilm development. Early-stage biofilm development of the wild-type IsoF, the *psoA* mutant PL11 and the non-motile strain GC25 was followed over time. Strains were transformed with plasmid pLUM3a, carrying a *psoA-gfp* transcriptional fusion. The activity of the *psoA* promoter was visualized by fluorescence microscopy at 30-min intervals. Owing to impaired motility of the strains, microcolonies of strains PL11 and GC25 were more compact, and contained a higher proportion of fluorescent cells than wild-type colonies. Scale bar, 5 μm.

To investigate whether, in addition to putisolvins, flagella may be required for cell migration out of microcolonies, we constructed a *fliM* mutant of *P. putida* IsoF, designated GC25, which no longer produces flagella and therefore is unable to swim. The microcolonies that strain GC25 formed were much more compact than the ones of the wild-type strain, suggesting that flagella are important for the positioning of cells within the aggregates (Fig. 5). Moreover, putisolvin producers migrated only at a very low rate out of the microcolonies when compared with the wild-type. These results show that the dissociation of cells from microcolonies is dependent on both flagella-driven motility and the production of putisolvin biosurfactants.

### Spatial expression of *psoA* in biofilms

We followed the temporal and spatial expression of putisolvins within biofilms of *P. putida* IsoF using the *P*_*psoA*_-*gfp* transcriptional fusion. Similar to our previous results (Fig. 1), we found that fluorescent cells were mainly located at the periphery or outside of microcolonies. In contrast, when the same transcriptional fusion was present in the putisolvin-defective mutant PL11 or the non-motile mutant GC25, we observed both a higher proportion of QS-induced cells and a more homogenous induction across cells, especially in GC25 (Fig. 5). These findings provide evidence that asocial cell movement out of the colony restricts cross-induction in the wild-type strain, while cross-induction becomes increasingly possible when cells are forced to stay together.

### Putisolvins are private and not public goods

**Figure 6:**
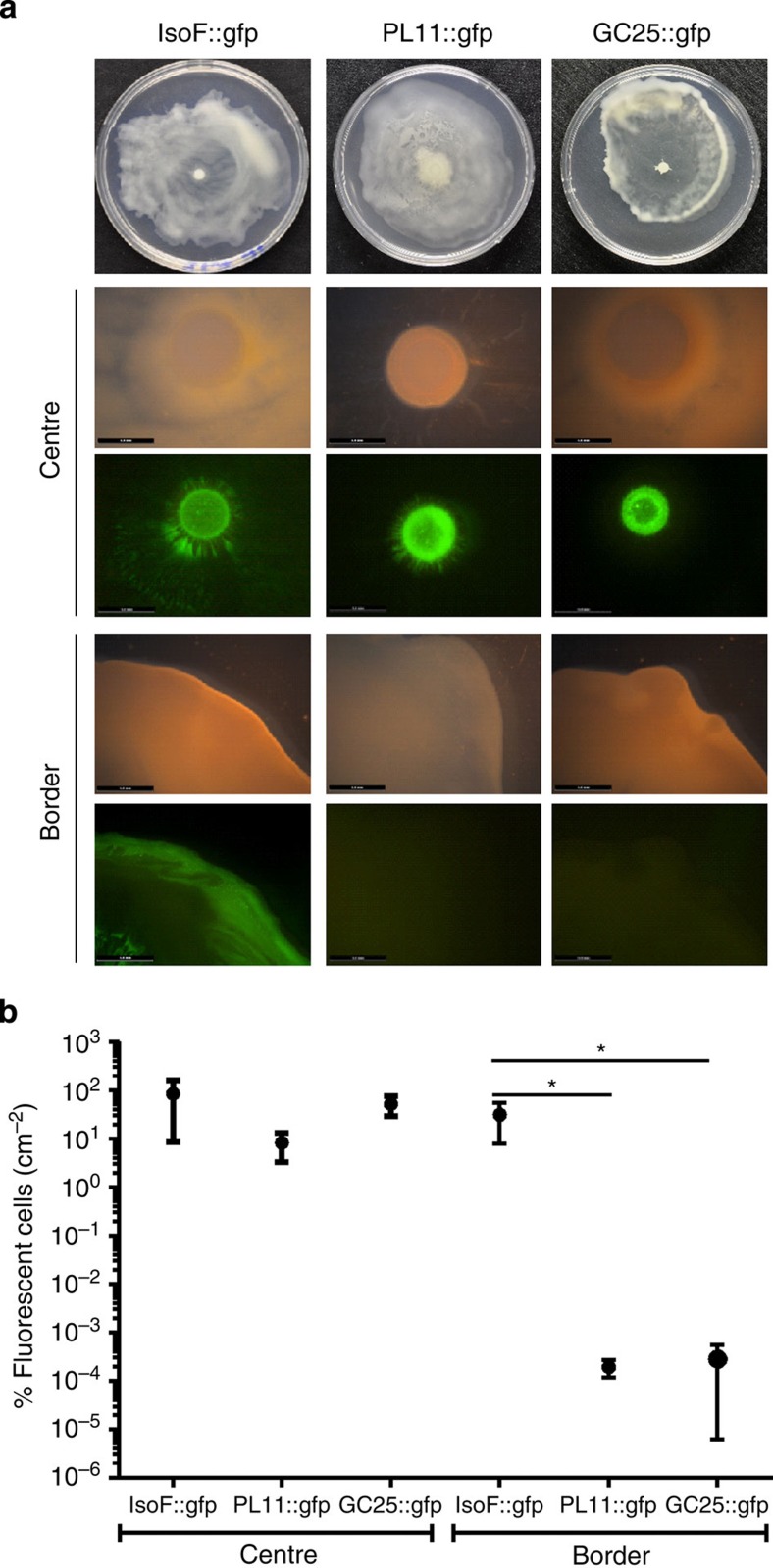
Putisolvins are not a public good in swarming colonies. Swarming plates were inoculated with equal amounts of the unmarked wild-type strain IsoF and GFP-marked derivatives of either IsoF, the putisolvin-deficient mutant PL-11 or the non-motile strain GC25. (**a**) Microscopic inspection revealed that the GFP-marked wild-type strain was present at the border of the swarm, while strains PL-11 and GC25 were mainly restricted to a region close to the inoculation point. Scale bar, 5 mm. (**b**) The dissemination of cells within the swarm was quantified by plating cells taken from the centre or the border region of the swarm on selective medium. Mean values of three independent experiments are shown with s.d. (one-way analysis of variance; *P<0.05).

A recent study in *P. aeruginosa* revealed that biosurfactants can represent public goods, which allow biosurfactant-defective mutants to swarm along with biosurfactant-producing wild-type cells^25^. In contrast, our single-cell analyses suggest that biosurfactants in *P. putida* IsoF remain associated with the bacterial cell surface, thereby triggering individual-based and not group-based swarming. To test this hypothesis, we mixed a putisolvin-deficient or a flagella-deficient mutant with the wild-type on swarming plates. In support of our hypothesis, we found that the mutants stayed close to the inoculation point, forming a small colony, whereas the wild-type swarmed over the entire plate (Fig. 6), with swarming behaviour being unaffected by the presence of another strain (Supplementary Fig. 3). This result held across a wide range of strain mixing ratios (Supplementary Figs 3 and 4). These findings demonstrate that putisolvins are private rather than public goods, which mostly adhere to the producing cells, and can therefore not be used by other cells for swarming.

## Discussion

Our work breaks with the central assumption that QS primarily represents a regulatory mechanism to coordinate cooperative behaviours among cells at high population density. Instead, we show that QS can also do the opposite: trigger uncoordinated self-directed behaviour at low cell density. Specifically, we found that in the early stages of biofilm development, AHL production occurred stochastically in only a fraction of cells. AHL production in these cells triggered the synthesis of putisolvins, biosurfactants that remain associated with the producer’s cell surface, thereby resulting in cells individually moving out of the microcolony. As this asocial motility removes individuals with the highest AHL production from the consortium, it exerts a negative feedback on cells left behind by delaying AHL cross-induction within the microcolony. It is important to note that we were only able to discover the lack of cross-induction and asocial motility, because AHLs were stochastically expressed at low population density. If all cells had started expressing AHLs at the same time, we would have erroneously concluded that putisolvin serves as a public good, coordinately expressed in the consortium to allow cooperative motility. This highlights that the mere observation of individual cells doing the same thing at the same time does not necessarily mean that coordination through communication and the sharing of pubic goods are involved^26^.

There are at least three reasons why stochasticity can arise in our system. First, Kaplan and Greenberg^27^ showed that AHL-dependent QS can be an extremely sensitive system, as demonstrated in *Vibrio fischeri*, where one to two AHL molecules per cell are sufficient to trigger autoinduction, suggesting that at very low AHL concentrations QS is intrinsically stochastic. Second, although the classic QS model assumes that AHLs are diffusing into the cell from the surroundings, such that the population density determines induction levels, we suggest that at the onset of QS the signal molecules are not released from the producing bacterium but directly bind to their cognate cytoplasmic receptors, which, as a consequence, results in self-induction of the cell’s QS cascade, and not in cross-induction. This possibility is especially probable in our study system, as the *P. putida* IsoF AHLs contain relatively long fatty acid chains, which often require transporters to be actively released from the cell^28^,^29^,^30^. Third, physiological differences between cells, particularly when grown as a biofilm, may exist that affect AHL production or the sensitivity of the QS response. Moreover, cross-induction seems to be additionally impeded in our system, because induced cells leave the consortium, which presumably results in reduced local AHL concentrations.

Although previous work has revealed heterogeneity in QS both at low and high cell densities, the situation described in this study is unique, as it is the first example that QS heterogeneity serves as a mechanism to trigger a self-directed behaviour of individual cells. At low cell density, heterogeneity in the initiation of QS has been observed in *P. aeruginosa* when single cells were confined in small volumes in a microfluidic device^31^. In this study, it is not only shown that QS induction is highly variable but also that low numbers of cells, even single cells, are able to initiate QS, supporting the idea of QS self-induction. At high cell density, meanwhile, Anetzberger *et al*.^32^ showed that the expression of QS-regulated bioluminescence in *Vibrio harveyi* is heterogenous. In a subsequent study, a working model was presented, in which the combination of the different *V. harveyi* signal molecules available (this organism produces three structurally unrelated signal molecules), rather than cell density *per se*, determines the timing of QS-regulated traits in this species^33^. Likewise, induction of AHL-controlled bioluminescence in individual *V. fischeri* cells was found to differ widely in time scale and in the overall intensity, suggesting that QS has relatively imprecise control over the response of an individual cell^34^. Although heterogeneity in QS seems frequent, a remaining key question is whether the heterogeneity is simply an inevitable outcome of the regulatory mechanism controlling AHL production, or whether it has an adaptive function. Although speculative at this stage, it seems plausible that in our case of asocial motility, leaving the microcolony can be beneficial under nutrient depletion. Even more so in mixed biofilms, where natural selection could favour individual-based early dispersal to reach new resources faster than the competitors^35^.

Although our data indicate that both AHL and putisolvin production is self-directed and thereby asocial during the early stages of biofilm formation, the pattern conceivably changed in older biofilms. Although AHL-producing cells first leave the microcolonies by themselves, we hypothesize that over time AHLs accumulate in the microcolonies, eventually leading to cross-induction of neighbouring cells. This idea is supported by the observation that *psoA* expression is more homogenous and increases faster in mutants impaired in motility when compared with expression of *psoA* in the wild-type background (Fig. 5). Furthermore, typical AHL cross-induction of cells has been observed in *P. putida* IsoF microcolonies grown in a microfluidic setup, in which motility of cells was restricted by coating the substratum with polylysin^36^. Thus, at later stages of biofilm development, AHL signalling eventually becomes a social trait, which then results in all cells producing putisolvins. This, in turn, leads to the hallmark of biofilm structural development in our setup, which was the sudden collapse of microcolonies at high cell densities. As nutrients are probably limiting at this point of biofilm development, even self-directed motility can become a social trait, because putisolvin-mediated biofilm dispersal will prevent overcrowding and allow the left behind population to resume growth. Indeed, the putisolvin-mediated microcolony collapse is reminiscent to the role of QS-controlled rhamnolipid production in detachment of cells from mature *P. aeruginosa* biofilms, which was suggested to release the stress arising from nutrient limitation at high population density^37^.

Our experiment showing that putisolvin-deficient mutants are unable to swarm with the wild-type strain demonstrates that putisolvins do not represent public goods (Fig. 6). In support of this, it has been shown that putisolvins preferentially adhere to the cell surface of the producing cell^19^. Interestingly, adhesion to bacterial cell surfaces has also been shown for other biosurfactants^38^,^39^,^40^, which may therefore represent a more general phenomenon. In analogy to our findings, Burch *et al*.^41^ showed that the biosurfactant syringafactin, which is produced by the plant epiphyte *P. syringae* pv. syringae B728a, is adsorbed to the adjacent waxy cuticle or retained on the bacterial cell surface. It is shown that its production primarily benefits the producer both by attracting moisture and facilitating access to nutrients.

Our results relate to recent work on QS in *P. aeruginosa*, where it has been shown that QS not only coordinates the expression of public goods at the group level, but also directs the expression of metabolically important enzymes at the cellular level^13^,^15^. The QS regulatory control over both social and self-directed traits has been interpreted as an adaptation to prevent invasion of cheating mutants. The idea is that the fitness increase a QS-deficient mutant gains by exploiting a QS wild-type strain, is cancelled by the fitness loss these mutants face, because they lack an important cellular enzyme. The situation is clearly different in our study system where the risk of cheating is reduced, because both the AHL-signal and putisolvins are not or only partially available to others. Taken together, our insights highlight that QS is much more complex than previously thought, as the traits being induced by QS can cover the entire continuum from a cooperative public good trait that generates benefits to others (for example, elastase production^11^), to extracellular traits that mostly generate self-directed benefits (for example, putisolvin production), to entirely intracellular traits that solely provide benefits to the producer.

## Methods

### Strains and culture conditions

Bacterial strains and plasmids used in this study are listed in Supplementary Table S1. *Escherichia coli* strains used for recombinant manipulations were propagated in Luria–Bertani medium at 37 °C. Plasmids were delivered to *P. putida* by triparental mating^22^. Briefly, donor, recipient and helper strain, *E. coli* HB101(pRK600), were harvested from overnight cultures, mixed and spot-inoculated on Luria–Bertani plates. After overnight incubation at 37 °C, transconjugants were isolated on Pseudomonas Isolation Agar (PIA) at 30 °C. *P. putida* strains were grown in modified AB medium supplemented with 10 mM sodium citrate^42^ (referred to as ABC medium). When required, media were supplemented with antibiotics at the following concentrations. For *E. coli*: 50 μg ml^−1^ ampicilin, 50 μg ml^−1^ kanamycin, 10 μg ml^−1^ gentamycin, 10 μg ml^−1^ tetracycline and 50 μg ml^−1^ trimethoprim. For *P. putida*: 100 μg ml^−1^ kanamycin, 20 μg ml^−1^ gentamycin and 100 μg ml^−1^ tetracycline.

### Construction of *P. putida* IsoF mutants

The *psoA* mutant PL11 was generated as follows: an internal *psoA* fragment was PCR amplified using the primers psoAF (5′- ctgatggtgtcgttcgaagagg -3′) and psoAR (5′- gctcgtcgagcacgtacaactg -3′). The amplicon was digested with SmaI and cloned into the gene replacement vector pEX18Gm cut with the same enzyme. The resulting plasmid, pEX18*psoA*, was mobilized into *P. putida* IsoF by triparental mating and gene replacement mutants were selected on PIA medium containing 20 μg ml^−1^ gentamycin. The *fliM* mutant GC25 was constructed by amplifying a *fliM* internal fragment using primers FliMF (5′- gccatggccgggttgaytc -3′) and FliMR (5′- gaygaygggctggtrcagac -3′), and cloning the PCR product blunt-ended into the Stul-digested gene replacement vector pSHAFT2Gm. The resulting plasmid, pSHAFT2*fliM*, was used to construct a *fliM* mutant as described for the *psoA* mutant. A *pso* conditional mutant was constructed as follows: first, the gentamycin resistance cassette from pBBR1MCS-5 was amplified using the primers genF (5′- gcagcaacgatgttacgcag -3′) and genR (5′- ttggtaccccgatctcggcttgaacg -3′), the amplicon was digested with XbaI and KpnI (restriction site underlined), and cloned into plasmid pSC200 cut with the same enzymes, yielding plasmid pSC200Gm. Next, a 590-bp fragment beginning at the start codon of *psoA* was amplified using primers pos2F (5′- tgcctgccgccgaaacctt -3′) and pos590R (5′- atctagagccagccaataatcgcggtc -3′), and the resulting DNA fragment was blunt ended with Klenow fragment and cloned into the filled-in NdeI site of pSC200Gm. This plasmid was mobilized from *E. coli* CC118 into *P. putida* IsoF by conjugation and the conditional mutant was selected on PIA medium supplemented with 20 μg ml^−1^ gentamycin. The genetic structures of all mutants constructed were confirmed by PCR and sequence analysis. The following primers were used: pSHAFT2F (5′- CGCTCTCGCGGCTTACGTTC -3′), pSHAFT2R (5′- AAGCCAGGGATGTAACGCACTG -3′), peX_F (5′- CACCGACAAACAACAGATAA -3′), peX_R (5′- CCCCAGGCTTTACACTTT -3′) pSC200end (5′- GTCATACTGGCCTCCTGATGTCGT -3′).

### Construction of transcriptional fusions

The pUT/mini-Tn*5*Km-based plasmid pPLlas^21^ was used to integrate the GFP-based AHL sensor into the chromosome of *P. putida* IsoF. Three independent mutants with different insertion positions were purified and used for flow cell experiments. To construct a *P*_*psoA*_-*gfp* transcriptional fusion, the *psoA* promoter region was PCR amplified using the primers p-psoAF (5′- aggatccgattctaagctttgcggcg -3′) and p-psoAR (5′- tggatccgctcagggcaaaggtttcg -3′). PCR fragments were cloned as BamHI fragments (restriction sites are underlined) into the respective site of the promoter–probe vector pGA-G1, generating the plasmids pPLM1 (*P*_*psoA*_-*gfp*). A P_*psoA*_*-cfp* fusion was generated by cloning the PCR product containing the *ecfp* gene from pBK-mini-Tn*7* into the TOPO vector. Then, the *psoA* promoter region from PLM1 was inserted as a BamHI fragment upstream of the *ecfp* gene in this plasmid. Finally, the the *P*_*psoA*_*-cfp* cassette was excised as a SacI fragment and inserted into the same site of plasmid pBBR1MCS-3, yielding pLUM3 (P_*psoA*_*-cfp*). The plasmids were mobilized from *E. coli* CC118 to *P. putida* strains by conjugation and selected on PIA medium supplemented with 50 μg ml^−1^ gentamycin (pPLM1) or 100 μg ml^−1^ tetracycline (pLUM3).

### Measurement of promoter activities

*P. putida* strains harbouring pPLM1 were grown in 10 mM ABC medium for 24 h at 30 °C with continuous shaking. When required, 3-oxo-C10 homoserine lactone was added to the medium at a final concentration of 5 μM. Green fluorescence was measured using 200 μl samples in a microtitre plate reader (SynergyTM HT, MWG Biotech, Germany) with an excitation wavelength of 485 nm and emission detection at 528 nm. The data were corrected for autofluorescence and processed with the KC4 software (BioTek Instruments). Specific fluorescence was calculated by normalizing relative fluorescence to OD_600_, which was simultaneously measured in the instrument.

### Swarming motility assays

Swarming motility was determined on ABC agar plates supplemented with 0.1% casamino acids and solidified with 0.4% (wt/vol) agar as described previously^43^. Briefly, overnight cultures were adjusted to an OD_600_ of 0.1, and 2 μl samples were inoculated on swarming plates, which were incubated for 3 days at 30 °C. The swarming plates were supplemented with 0.5%, 1% or 2% (wt/vol) rhamnose when appropriate.

### Biosurfactant production

Semi-quantitative measurement of biosurfactant activity was done by using the drop-collapsing assay, in which the reduction of surface tension causes a collapse of the droplet placed on a hydrophobic surface. To quantify biosurfactant production, the decrease of surface tension between culture medium and air was determined with a Du Nouy ring^18^.

### Cultivation and analysis of biofilms

Biofilms were grown in flow cells supplied with ABC medium. The flow system was assembled and prepared as described previously^22^. Briefly, the flow channels were inoculated with *P. putida* cultures grown for at least five generations at a low cell density (OD_600_<0.3) in minimal medium supplemented with citrate as the carbon source. The medium flow was kept at a constant rate of 0.2 mm s^–1^ by a Watson–Marlow 205S peristaltic pump. The incubation temperature was 30 °C. Microscopic inspection and image acquisition were performed using a confocal laser scanning microscope (DM5500Q; Leica) equipped with a × 40/1.3 or a × 63/1.4 oil objective. Captured images were analysed with the Leica Application Suite (Mannheim, Germany) and the Imaris software package (Bitplane, Switzerland). Images were prepared for publication using CorelDraw (Corel Corporation) and PowerPoint (Microsoft) software.

To quantify the proportion of AHL-induced and non-induced cells, five independent experiments were conducted. In each experiment, five random positions were chosen on the flow chamber glass surface and surveyed every 30 min for 10 h, starting 6 h post inoculation. Aliquots of a low-cell-density inoculum (OD_600_=0.01) were used to initiate the flow cell biofilms to allow single cell analysis. To distinguish between free and colony-associated cells, an aggregate size of eight cells was defined as threshold, below which cells were considered as free. Using this threshold, the average aggregate size for free cells was found to be 3.5±0.5. As transmitted light was used to obtain the total cell number, only microcolonies with few cell layers were used for quantification. When analysing older biofilms consisting of multiple cell layers, we either used strains marked with mCherry or stained cells with SYTO 62 (Life Technologies).

### Statistical analysis

An LMM was used to test whether the proportion of AHL-induced cells differs between free and colony-associated cells, and whether the induction pattern changes over time. Position identity within experiments was introduced into the model as a random factor to account for the nested approach (that is, five positions within five experiments). Prism (GraphPad Software) was used for one-way analysis of variance. If the analysis of variance yielded significant differences between factor levels, the Bonferroni method was applied for pairwise comparisons between factor levels.

## Author contributions

L.E. designed the research; G.C.-O. and P.L. performed the experiments and contributed equally to the study. All authors analysed the data and contributed to the writing of the paper.

## Additional information

**How to cite this article:** Cárcamo-Oyarce, G. *et al*. Quorum sensing triggers the stochastic escape of individual cells from *Pseudomonas putida* biofilms. *Nat. Commun.* 6:5945 doi: 10.1038/ncomms6945 (2015).

## Supplementary Material

Supplementary Information Supplementary Figures 1-4, Supplementary Table 1, and Supplementary References
