# Supplementary material for: Quorum sensing triggers the stochastic escape of individual cells from Pseudomonas putida biofilms
Source: Nat Commun. 2015 Jan 16;6:5945. doi: 10.1038/ncomms6945 (PMC4309448; doi:10.1038/ncomms6945)
Supplement: Supplementary Information — Supplementary Figures 1-4, Supplementary Table 1, and Supplementary References [file ncomms6945-s1.pdf]

Supplementary Information

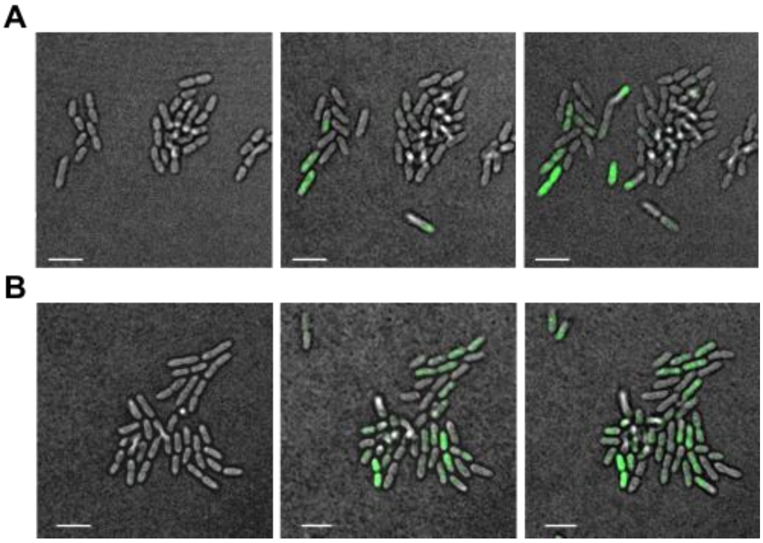

Supplementary Fig. 1. Exposure to saturating concentration of AHL does not affect stochastic AHL-induction at early stages of biofilm development of *P. putida* IsoF. Flow cells were initiated with IsoF carrying the GFP-based AHL sensor**.** (A) After microcolonies were formed, only a few cells start to produce AHLs. Furthermore, the appearance of induced-single-cells in the proximity of microcolonies suggests they left the colony once activated. (B) When the experiment was performed under uniform amount of autoinducer in the medium, 0.5 µM 3-oxo-C10-HSL, a similar pattern in terms of induction heterogeneity was observed, although induction occurred earlier and in a higher proportion of cells. Scale bar: 5 µm.

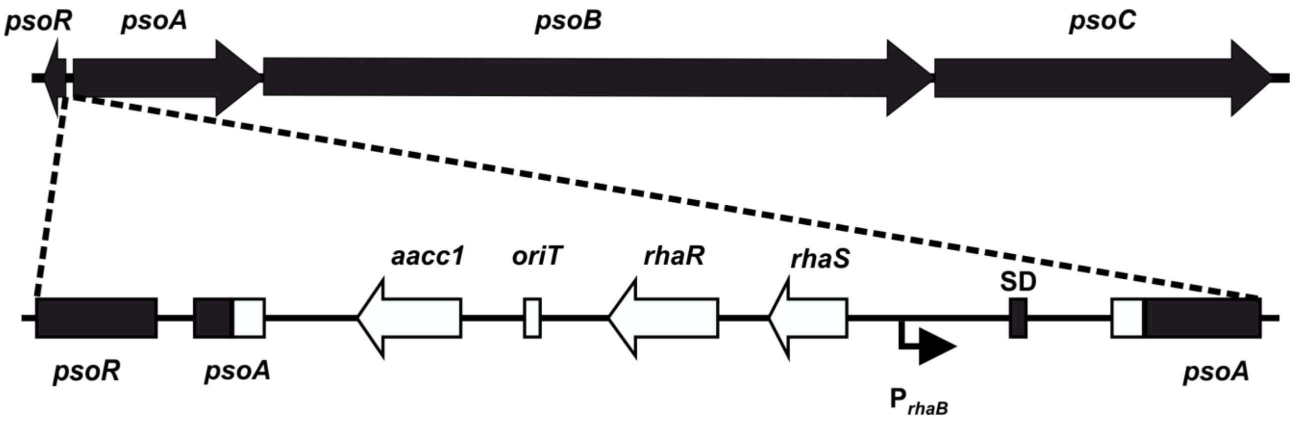

Supplementary Fig. 2. Structure of putisolvin biosynthetic cluster and schematic outline of the construction of the conditional *psoABC* mutant PL2, in which natural *psoA* promoter has been replaced by the rhamnose-inducible promoter $P_{rhaB.}$ In addition, the strain carries genes required for the activation of the P_{rhaB} promoter (*rhaS* and *rhaR*); aacC1, aminoglycoside acetyltranferase (gentamycin resistance); *oriT*, origin of transfer; SD, Shirne-Delgarno.

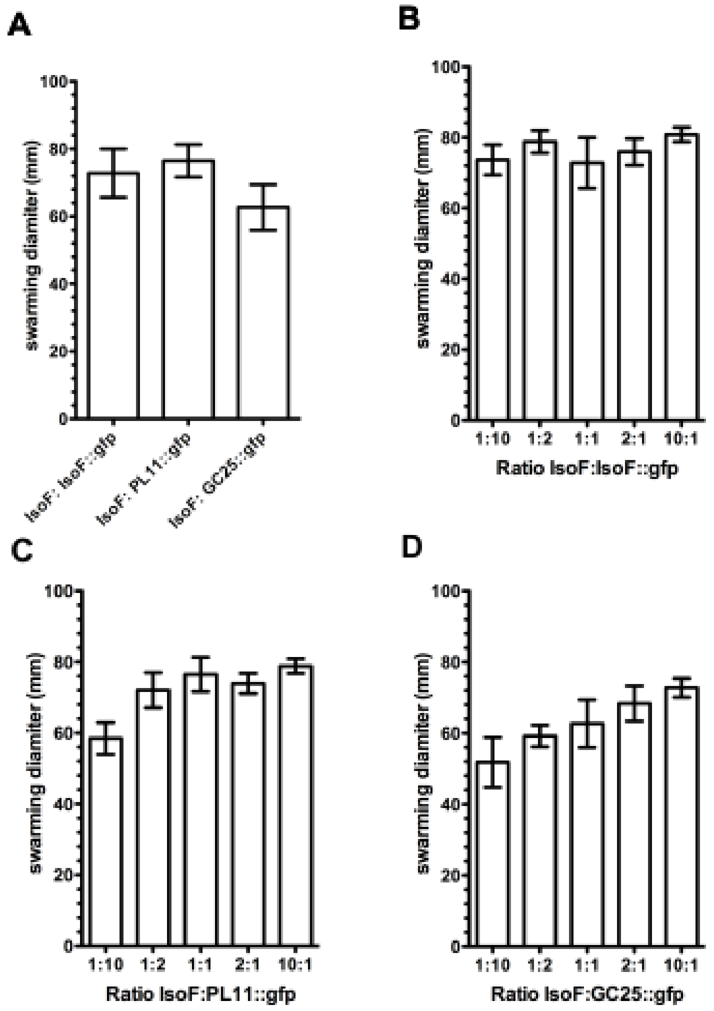

Supplementary Fig. 3. Swarming motility of mixed cultures. The wild type (IsoF) was combined with itself, the putisolvin-deficient PL11, or the flagella-deficient mutant GC25 strain on swarming plates. *(A)* In 1:1 mixes, the displacement of the swarming colony remains unaffected irrespective of the strain mixture. When mixing IsoF at different ratios with: (*B*) itself, IsoF::gfp; (*C*) with PL11::gfp; or (*D*) with GC25::gfp, the swarming performance was unaffected across most mixing ratios. Only when the proportion of IsoF was decreased to 10%, swarming displacement is slightly reduced in mixtures with PL11::gfp and GC25::gfp. These findings support our conclusions that PL11::gfp and GC25::gfp cannot swarm along with IsoF wild type. Mean values of three independent experiments are shown with s.e.m.

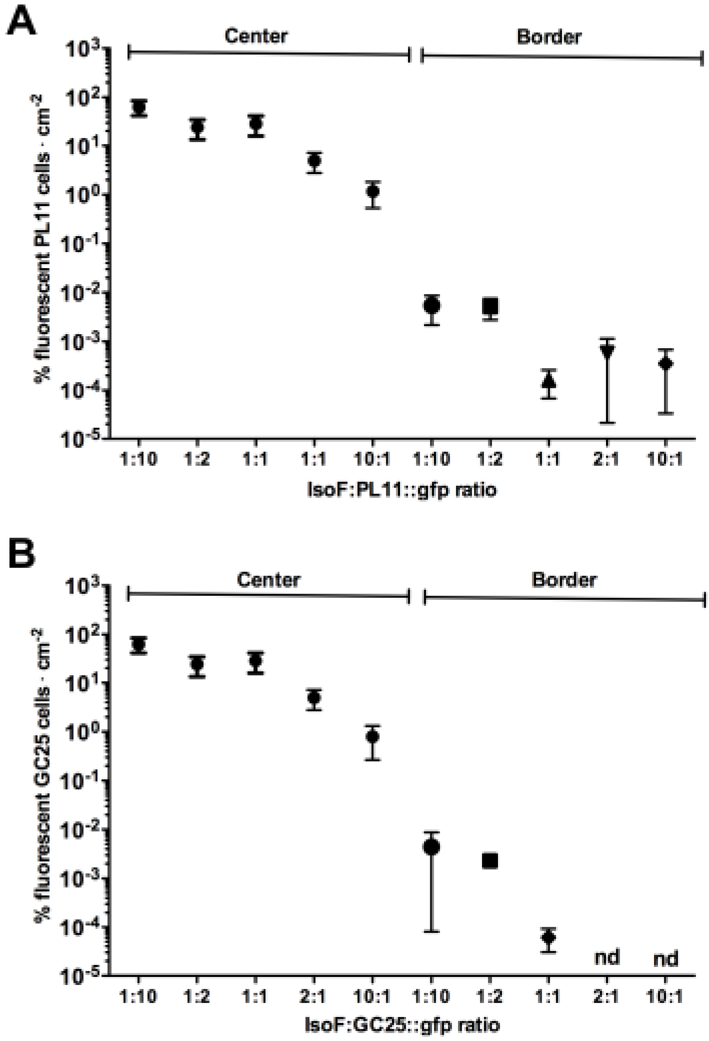

Supplementary Fig. 4. Putisolvins are not public goods in swarming colonies. In mixed colonies with IsoF, mutants deficient for (*A*) putisolvin (PL11:gfp) and (*B*) flagella (GC25:gfp) production remain located in the center of the plate, while IsoF swarms to the edges. This pattern remained consistent across a wide range of mixing ratios, confirming that putisolvin is a private good under swarming conditions. Mean values of three independent experiments are shown with s.e.m.

Supplementary Table 1. Bacterial strains and plasmids used in this study

Strain or plasmid	Relevant properties	Source or reference
E. coli		
MT102	*araD139* (*ara*-leu)7679 Δ*lac thi hsdR*	This Laboratory
CC118(λpir)	Δ(*ara-leu*) *araD* Δ*lacX74 galE galK phoA20 thi-1 rpsE rpoB argE*(Am) *recA1* λ*pir* lysogen	1
HB101	*recA thi pro leu hsd* M^+; Sm^R	2
P. putida		
IsoF	wild type; isolated from tomato roots; AHL^+	3
F117	*ppuI* mutant of IsoF; Km^R	4
GC3	*ppuR* mutant of IsoF; Km^R	This Laboratory
PL11	*psoA* mutant of IsoF; Gm^R	This study
GC25	*fliM* mutant of IsoF; Gm^R	This study
PL2	conditional *psoA* mutant of IsoF; P_{rhaB}- *psoA*; Gm^R	This study
IsoF-mcherry	IsoF tagged with mcherry, Gm^R	This laboratory
**Plasmids**		
pEX18	gene replacement vector; Gm^R	5
pEX18psoA	pEX18 containing an internal fragment of *psoA* in the SmaI site; Gm^R	This study
pSHAFT2	pUTmini-Tn*5*Cm with deleted BglII fragment; contains the *tnp* gene and I end of mini-Tn*5*; Gm^R	This laboratory
pSHAFT2fliM	pSHAFT2 containing an internal fragment of *fliM* in the StuI site; Gm^R	This study
pSC200	*ori*R6K; rhamnose-inducible promoter P_{RhaB}; Tp^R	6
pSC200Gm	Gm^R resistance cassette cloned into XbaI/KpnI sites of pSC200; Gm^R	This study
pPO1	pSC200Gm carrying internal *psoA* fragment; Gm^R	This study
pPLlas	pUT/mini with Km^r::*lasR*-P_{lac}-P_{lasB}-*gfp*(ASV)-T_0-T_1 in the NotI site	7
pRPL4las	RP4 with Km^R::*las*R-P_{lac}-P_{lasB}-*gfp*(ASV)	7
pGA-G1	Promoter probe vector carrying a promoterless *gfp*-mut3 gene; Gm^R	8
pLUM1	P_{psoA} promoter cloned into pGA-G1; Gm^R	This study
pLUM3	pBBR1MCS-3 carrying a P_{psoA}::*cfp* fusion; Tc^R	This study
pBAH8	$P_{A1/04/03}$-*gfp*mut3 fusion in pBBR1MCS-2; Km^R	9
pBBR1MCS-3	Broad-host-range plasmid, *lac*Zα; Tc^R	10
pBBR1MCS-5	Broad-host-range plasmid, *lac*Zα; Gm^R	10
pRK600	ColE1 RK2-Mob^+ RK2-Tra^+; helper plasmid; Cm^R	11

References

1 Herrero, M., de Lorenzo, V. & Timmis, K. N. Transposon vectors containing non-antibiotic resistance selection markers for cloning and stable chromosomal insertion of foreign genes in gram-negative bacteria. *J Bacteriol* **172**, 6557-6567 (1990).

2 Boyer, H. W. & Roulland-Dussoix, D. A complementation analysis of the restriction and modification of DNA in Escherichia coli. *J Mol Biol* **41**, 459-472 (1969).

3 Steidle, A. *et al.* Visualization of N-Acylhomoserine Lactone-Mediated Cell-Cell Communication between Bacteria Colonizing the Tomato Rhizosphere. *Applied and Environmental Microbiology* **67**, 5761-5770, doi:10.1128/AEM.67.12.5761-5770.2001 (2001).

4 Steidle, A. *et al.* Identification and characterization of an N-acylhomoserine lactone-dependent quorum-sensing system in Pseudomonas putida strain IsoF. *Applied and Environmental Microbiology* **68**, 6371-6382 (2002).

5 Hoang, T. T., Karkhoff-Schweizer, R. R., Kutchma, A. J. & Schweizer, H. P. A broad-host-range Flp-FRT recombination system for site-specific excision of chromosomally-located DNA sequences: application for isolation of unmarked Pseudomonas aeruginosa mutants. *Gene* **212**, 77-86 (1998).

6 Ortega, X. P. *et al.* A putative gene cluster for aminoarabinose biosynthesis is essential for Burkholderia cenocepacia viability. *J Bacteriol* **189**, 3639-3644, doi:10.1128/JB.00153-07 (2007).

7 Lumjiaktase, P., Aguilar, C., Battin, T., Riedel, K. & Eberl, L. Construction of self-transmissible green fluorescent protein-based biosensor plasmids and their use for identification of N-acyl homoserine-producing bacteria in lake sediments. *Appl Environ Microbiol* **76**, 6119-6127, doi:10.1128/AEM.00677-10 (2010).

8 Schwager, S., Lumjiaktase, P., Stöckli, M., Weisskopf, L. & Eberl, L. The genetic basis of cadmium resistance of Burkholderia cenocepacia. *Environmental Microbiology Reports* **4**, 562-568, doi:10.1111/j.1758-2229.2012.00372.x (2012).

9 Huber, B. *et al.* Genetic analysis of functions involved in the late stages of biofilm development in Burkholderia cepacia H111. *Mol Microbiol* **46**, 411-426 (2002).

10 Kovach, M. E. *et al.* Four new derivatives of the broad-host-range cloning vector pBBR1MCS, carrying different antibiotic-resistance cassettes. *Gene* **166**, 175-176 (1995).

11 de Lorenzo, V. & Timmis, K. N. Analysis and construction of stable phenotypes in gram-negative bacteria with Tn5- and Tn10-derived minitransposons. *Methods Enzymol* **235**, 386-405 (1994).
